# Synthesis, Characterization and *in Vitro* Evaluation of Manganese Ferrite (MnFe_2_O_4_) Nanoparticles for Their Biocompatibility with Murine Breast Cancer Cells (4T1)

**DOI:** 10.3390/molecules21030312

**Published:** 2016-03-11

**Authors:** Samikannu kanagesan, Sidek Bin Ab Aziz, Mansor Hashim, Ismayadi Ismail, Subramani Tamilselvan, Noorjahan Banu Binti Mohammed Alitheen, Mallappa Kumara Swamy, Bandaru Purna Chandra Rao

**Affiliations:** 1 Materials Synthesis and Characterization Laboratory (MSCL), Institute of Advanced Technology (ITMA), Universiti Putra Malaysia, UPM Serdang 43400, Selangor, Malaysia; sidekaziz@gmail.com (S.A.B.A.); mansorhashim@gmail.com (M.H.); kayzen@gmail.com (I.I.); 2 Department of Physics, Faculty of Science, Universiti Putra Malaysia, UPM Serdang 43400, Selangor, Malaysia; 3 Department of Cell and Molecular Biology, Faculty of Biotechnology and Biomolecular Sciences, Universiti Putra Malaysia, Serdang 43400, Selangor, Malaysia; drstamilselvan@gmail.com (S.T.); noorjahan@upm.edu.my (N.B.B.M.A.); swamy.bio@gmail.com (M.K.S.); 4 Department of Crop Science, Universiti Putra Malaysia (UPM), Serdang 43400, Selangor, Malaysia; 5 Department of Applied Science and Humanities, Sasi Intitute of Technology and Engineering, Tadepalligudem, West Godavari District 534101, Andhra Pradesh, India; nanopurna@gmail.com

**Keywords:** manganese ferrite, nanoparticles, magnetization, breast cancer, cytotoxicity

## Abstract

Manganese ferrite (MnFe_2_O_4_) magnetic nanoparticles were successfully prepared by a sol-gel self-combustion technique using iron nitrate and manganese nitrate, followed by calcination at 150 °C for 24 h. Calcined sample was systematically characterized by X-ray diffraction (XRD), Fourier transform infrared spectroscopy (FTIR), transmission electron microscopy (TEM), and vibrational sample magnetometry (VSM) in order to identify the crystalline phase, functional group, morphology, particle size, shape and magnetic behavior. It was observed that the resultant spinal ferrites obtained at low temperature exhibit single phase, nanoparticle size and good magnetic behavior. The study results have revealed the existence of a potent dose dependent cytotoxic effect of MnFe_2_O_4_ nanoparticles against 4T1 cell lines at varying concentrations with IC_50_ values of 210, 198 and 171 μg/mL after 24 h, 48 h and 72 h of incubation, respectively. Cells exposed to higher concentrations of nanoparticles showed a progressive increase of apoptotic and necrotic activity. Below 125 μg/mL concentration the nanoparticles were biocompatible with 4T1 cells.

## 1. Introduction

Nowadays, research on magnetic ferrite nanoparticles has become of great interest due to their extraordinary magnetic properties and they have been used technologically in permanent magnets, ferrofluids, broad band transformers, magnetic sensors, *etc.* Special attraction has also been paid to biomedical applications like drug delivery, biosensors, magnetic resonance imaging and magnetic hydrothermia [1,2,3,4,5,6,7]. Manganese ferrite nanoparticles exhibit superior mechanical, luminescent and magnetic properties compared to other existing magnetic ferrite nanoparticles. In recent times, manganese ferrite nanoparticles synthesized by traditional ceramic methods [8,9,10] suffered from drawbacks like uncontrolled particle size, uniformity, poorly defined stoichiometric composition, the presence of impurities during ball milling, chemical inhomogeneity, contamination, and high calcination temperatures [11,12]. Many preparation methods have been used across the globe in the process of synthesis of these magnetic nanoparticles, include sol-gel [13,14], flash combustion [15] citrate gel [16], co-precipitation [17,18], hydrothermal synthesis [19], sol-gel auto combustion [20], micro-emulsion [21] and low temperature combustion methods [22]. It was reported that the chemical routes are the most suitable to synthesize nanomagnetic particles, among them, the sol-gel self-combustion method has attracted considerable attention and said to be a flexible method to synthesize spinel MnFe_2_O_4_ nanoparticles. Researchers [19,20,21,23] have dedicated their efforts to the synthesis and study of spinel ferrites due to the special properties they exhibit in the nano range.

In recent years, various types of nanoparticles synthesized from inorganic as well as organic materials have shown potential applications in cancer therapy [24,25]. Magnetic nanoparticles used as drug delivery structures appear very beneficial as they show remarkable heating effects and thus provide an opportunity to target tumor cells specifically [26,27]. Most of the drugs used for treating cancer exhibits toxicity to both tumor and normal cells, causing side effects and this restricts the effectiveness of chemotherapy treatments. Therefore, understanding these nanoparticles and their toxicity is very important. In the past, though few researchers have studied the cytotoxic effects of different magnetic nanoparticles, their studies are restricted to only few magnetic nanoparticles [26,28,29]. Previously, a study by [28] showed that MnFe_2_O_4_nanoparticles of size 40nm were efficiently internalized by PC-12 cells, which suggest the possible use of these nanoparticles as an anticancer drug. However, there is a need to screen these nanoparticles before clinically used for cancer therapy. Therefore, this study was aimed at characterizing the structural, morphological, magnetic properties of MnFe_2_O_4_ nanoparticles synthesized by sol-gel self-combustion technique. Also, MnFe_2_O_4_ nanoparticles were evaluated for their cytotoxicity against 4T1 murine breast cancer cell lines.

## 2. Results and Discussion 

### 2.1. Fourier Transform Infrared Spectroscopy

**Figure 1:**
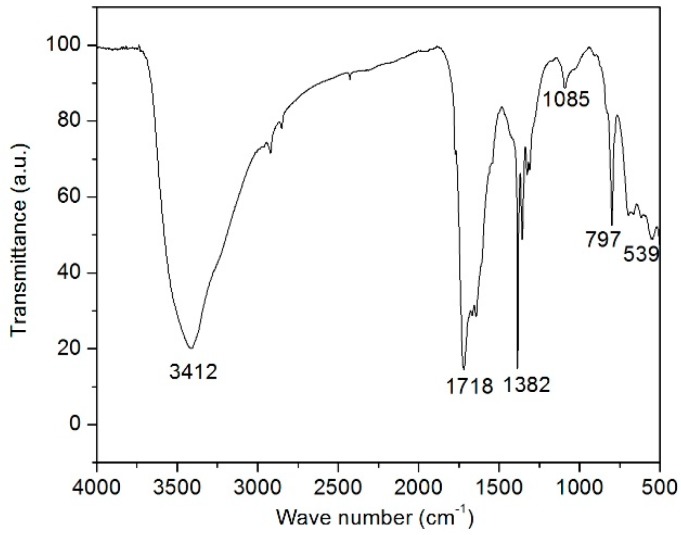
FT-IR spectra of manganese ferrite nano powder.

Figure 1 shows the FTIR spectrum of the calcined MnFe_2_O_4_ magnetic nanoparticles in the range between 500 and 4000 cm^−1^.

A broad band absorption peak appeared at 3412 cm^−1^ and a high frequency absorption peak was detected at 1718 cm^−1^, confirming the presence of O-H groups in the sample. The characteristic band at 1382 cm^−1^ is related to the symmetric vibrations of the NO_3_^−^ group [30]. Generally, the metal oxide vibrations occur below 1000 cm^−1^. The peaks appearing below 700 cm^−1^ are due to the spinel structure. The band around 539 cm^−1^ is attributed to the intrinsic vibrations of octahedral coordinated metal ions in the spinel structure, confirming that the prepared samples are spinel in structure [30].

### 2.2. XRD Analysis

**Figure 2:**
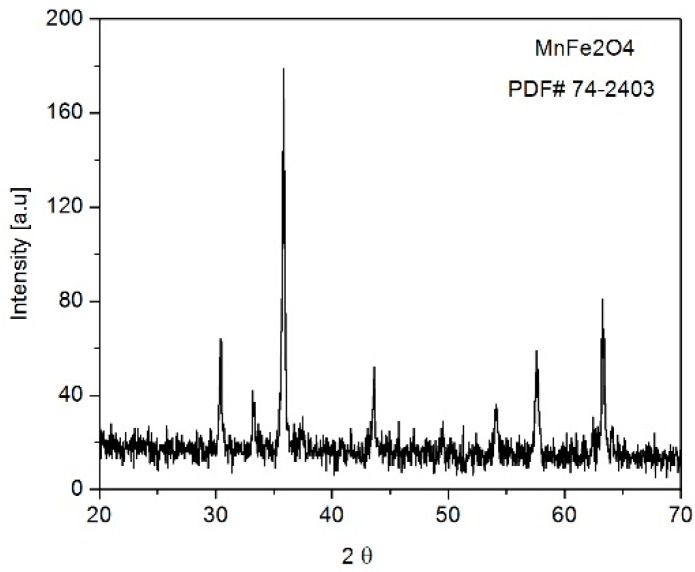
XRD pattern of manganese ferrite powder calcined at 150 °C for 24 h.

The X-ray diffraction pattern of a MnFe_2_O_4_ calcined sample is illustrated in Figure 2. All diffraction peak positions and relative intensities correspond to the Fd3m space group with a cubic structure which exactly coincides with the standard spinel manganese ferrite (JCPDS card no. 74-2403). The average crystallite size of MnFe_2_O_4_ was calculated by considering the full width at half-maximum (FWHM) of diffraction based on the Scherrer’s formula:

$$D=\frac{\left(0.9\lambda \right)}{\beta \cos\left(\theta \right)}$$

where *D* is the average particle size of the crystallites, λ is the incident wavelength, θ is the Bragg angle and β is the diffracted full width at half maximum (in radians) caused by crystallation. The average crystallite size of the resulting nanoparticles was 32 nm. It is interesting to note that spinel ferrite diffraction peaks were quite broad due to the small particle size. 

### 2.3. Morphological Analysis

The preparation process was clearly demonstrated to have a considerable influence on the morphologies of the resulting spinel ferrites.

**Figure 3:**
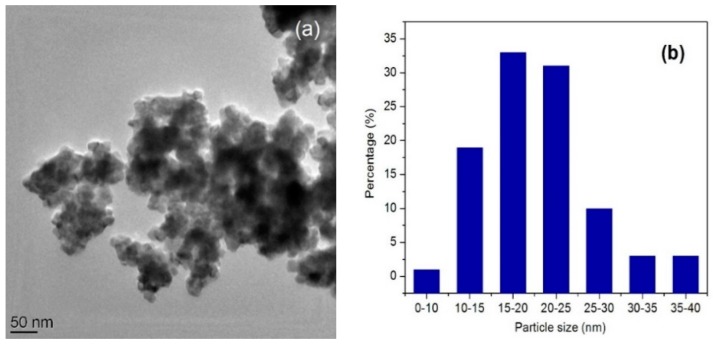
TEM image of calcined powder (**a**) and the corresponding particle size histogram (**b**).

The size, shape, and morphologies of the low-temperature synthesized MnFe_2_O_4_ nanoparticles were further determined by TEM. The TEM images (Figure 3) show that the majority of the particles are almost spherical in shape and agglomerated. The average particle size varied in the 25–35 nm range. The average particle size obtained from TEM is thus in good agreement with that determined from the Scherrer formula.

### 2.4. Magnetic Analysis

**Figure 4:**
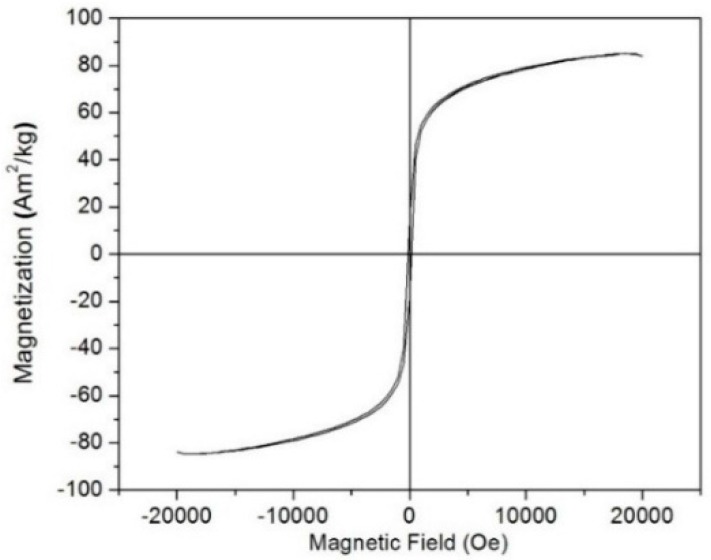
Hysteresis curve of manganese ferrite sample at room temperature.

Figure 4 presents the hysteresis loop of the calcined MnFe_2_O_4_ sample measured in a magnetic field by a vibrating sample magnetometer. The calcined MnFe_2_O_4_ powder exhibits ferromagnetic behavior with saturation magnetization (Ms), coercivity (Hc) and remanent magnetization (Mr), values of about 85 Am^2^/kg, 148.7 Oe, and 16.68 Am^2^/kg, respectively, and these values are varied from reported hydrothermally synthesized MnFe_2_O_4_ (Ms = 78.3 Am^2^/kg, Mr = 5.32Am^2^/kg, Hc = 45 Oe) [31] and the sample prepared by combustion route (Ms = 80 Am^2^/kg, Mr = 50Am^2^/kg, Hc = 85Oe) [32]. Generally, magnetic properties vary with the variations in the particle size, shape, crystallinity *etc.* The saturation magnetization value is almost equal to the reported bulk MnFe_2_O_4_ (73.8 Am^2^/kg, 80 Am^2^/kg) [31,32].

### 2.5. Cytotoxicity Analysis

The cytotoxicity of MnFe_2_O_4_ nanoparticles synthesized by the sol-gel salt combustion technique was evaluated against a murine breast cancer cell line (4T1) using the MTT assay. 

**Figure 5:**
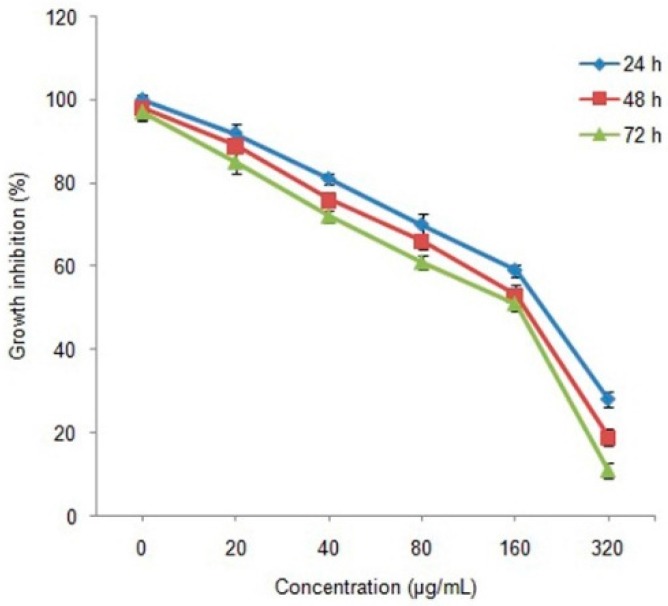
Growth inhibition of 4T1 murine breast cancer cells exposed to synthesized MnFe_2_O_4_ nanoparticles for 24, 48 and 72 h in cytotoxicity study using MTT assay.

**Figure 6:**
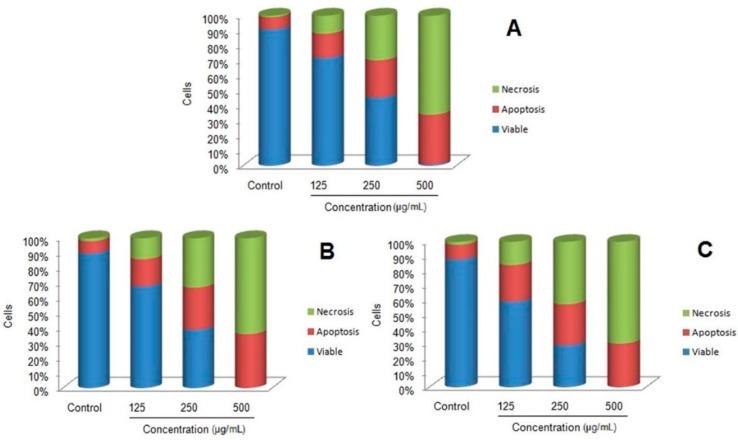
Flow cytometry analysis of untreated and treated 4T1 cells with MnFe_2_O_4_ nanoparticles for 24 h (**A**); 48 h (**B**) and 72 h (**C**) stained with acridine orange and propidium iodide (AO/PI).

The results of our study revealed the existence of a potent toxicity effect of MnFe_2_O_4_ nanoparticles against 4T1 cell lines at varying concentrations (Figure 5). The cell viability percentage was observed to decrease with increased concentration of MnFe_2_O_4_ nanoparticles indicating a dose dependent cytotoxic effect. With the increase of exposure time from 24 h to 72 h, the cell viability percentage was further reduced, irrespective of the concentration tested. After 24 h of incubation, control treatments showed the highest cell viability percentage (100%) while the lowest (11%) was observed at 320 μg/mL nanoparticle concentration after 72 h. IC_50_ values of 210 μg/mL, 198 μg/mL and 171 μg/mL were observed after 24, 48 and 72 h of incubation, respectively. Similarly, it has been reported that Mn nanoparticles induce time and dose dependent cytotoxic activity against N27 dopaminergic neuronal cells [33]. Further confirmation of the cell toxicity effect due to MnFe_2_O_4_ nanoparticles involving apoptotic changes and condensation of nuclei was studied using the AO/PI staining method. All viable and early apoptotic cells are stained by AO and generate green fluorescence, while necrotic and dead cells stained by PI produce red fluorescence. Our study revealed that increased exposure to MnFe_2_O_4_ nanoparticles concentrations over all incubation periods resulted in decreased cell viability in comparison to control cells (Figure 6).

**Figure 7:**
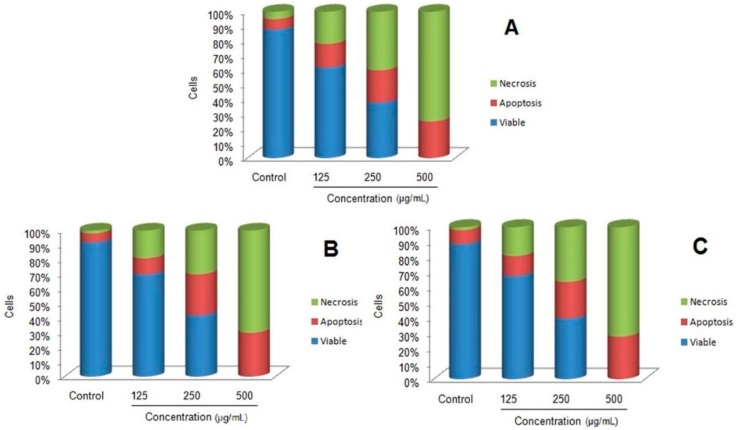
Flow cytometry analysis of untreated and treated 4T1 cells with MnFe_2_O_4_ nanoparticles for 24 h (**A**); 48 h (**B**) and 72 h (**C**) stained with annexin V-FITC/propidium iodide (PI).

When the cells were exposed to higher concentrations of nanoparticles they showed a progressive increase of apoptotic and necrotic activity. However, at 500 μg/mL 100% cytotoxicity with higher necrotic cells was observed after 48 h and 72 h of incubation. The results showed 36% of apoptotic cells and 64% of necrotic cells after 48h of treatment at 500 μg/mL while, at the same concentration 30% of apoptotic cells and 70% necrotic cells were observed after 72 h. This clearly suggests that higher concentrations of MnFe_2_O_4_ nanoparticles cause more necrotic activity than apoptosis. The induction of apoptotic cellular death observed in 4T1 cell lines was further quantified by using flow cytometry after staining the cells with green fluorescent FITC labeled Annexin V dye because of its sensitivity [34]. In this method, annexin V attached to the externalized residues of phosphatidylserine occurring on the cell membrane of apoptotic cells at their early stage are visualized using fluorescence microscopy [24,35]. Flow cytometric data of 4T1 cell lines treated with MnFe_2_O_4_ nanoparticles stained with annexin V-FITC/propidium iodide are shown in the Figure 7. The results clearly indicate the dose dependent activity of cell toxicity with 100% cell death observed at 500 μg/mL concentration under all incubation period while, apoptotic and necrotic cell population increased with higher concentrations of nanoparticles treatments. The cells treated at 500 μg/mL concentration for 24, 48 and 72 h showed 25%, 30% and 28% of apoptosis, respectively. Conversely, at 500 μg/mL concentration for 24, 48 and 72 h showed 75%, 70% and 72% of necrotic activity, respectively. Similarly, dose dependent cytotoxicity with increased apoptotic and necrotic cells were observed in 4T1 cells when treated with copper ferrite nanoparticles [23].

Previous studies have documented that metal nanoparticles produce cellular damage and apoptosis due to induced oxidative stress, inflammatory responses triggered by reactive oxygen species and increased levels of TNF-α. According to Park *et al.* [36], the cytotoxicity is attributable to the ionization of metallic nanoparticles in cells as expressed by a “Trojan-horse” type mechanism. Based on these reports it can be affirmed that MnFe_2_O_4_ nanoparticles after entering the cells release their ions and cause various cellular alterations/damages leading to cell death.

From the present study, it is observed that MnFe_2_O_4_ nanoparticles were efficient in inducing cytotoxicity against 4T1 cells with a dose dependent apoptotic and necrotic activity. However, higher concentrations were more detrimental to the cell survivability as they induced more necrosis. In humans, cell death due to apoptosis or necrosis is an important biological process and any abnormalities in cell death may result in various diseases including cancer. Thus, a better understanding of apoptosis and necrosis processes can be a target for treatment [37,38]. Presently, cytotoxic drugs are mostly targeted on inducing apoptosis in cancer cells, however resistance to apoptosis, apoptosis in normal cells associated with side effects leads to treatment failure. Despite continuing progress made in the discovery cytotoxic agents, there is a need to explore new key molecules [38]. In this regard, the results of our study are promising and there MnFe_2_O_4_ nanoparticles can serve as a potential cytotoxic agent for various targeted therapies including cancer treatment.

## 3. Experimental Section

### 3.1. Preparation of MnFe_2_O_4_ Nanoparticles

AR grade manganese nitrate, ferric nitrate and citric acid were purchased from Aldrich Chemicals (St. Louis, MO, USA). Manganese nitrate and ferric nitrate in the molar ratio of 1:2, and citric acid in the ratio of 1:1 with nitrates were dissolved in a minimum amount of ethanol. A suitable amount of oleic acid was added to the solution. The solution was stirred for 4 h at room temperature and concentrated in a vacuum rotary evaporator at 60–80 °C to remove surplus water. The gel was heated at 150 °C in a hot air oven for 24 h. A brown color MnFe_2_O_4_ powder was obtained.

### 3.2. Characterization

The X-ray diffraction patterns of the calcined powder sample was studied by a PAN Analytical (PANalytical B.V.Corporate Marketing and Communications Department Lelyweg 1, 7602 EA PO Box 13, 7600 AA, Almelo, The Netherlands) X’pert pro X-ray diffractometer with CuKa radiation at 45 kV, 40 mA (k = 0.15406 nm) and fixed 2θ is in the range of (20° ≤ 2θ ≤ 70°). The microstructure observation of the specimen was performed by transmission electron microscopy (JEM 3010-JEOL, Sollentuna, Sweden) with an accelerating voltage of 200 kV. The magnetic characteristics of the specimen were measured at room temperature using vibrating sample magnetometry (LakeShore 7407, Lake Shore Cryotronics, Inc., 575 McCorkle Blvd, Westerville, OH, USA).

### 3.3. Evaluation of Cytotoxicity Activity

We evaluated the cytotoxic effect of MnFe_2_O_4_ nanoparticles on the murine breast cancer cell line (4T1) as described previously by Swamy *et al.* [25]. Briefly, the cells were cultured on Dulbecco’s Modified Eagle’s Medium (DMEM) added with l-glutamine (2 mM), penicillin (100 U/mL), streptomycin (100 g/mL) and fetal bovine serum (10%). Approximately 5 × 10^4^ cells were inoculated in each well of 96-well plates and incubated in a carbon dioxide incubator maintained at 37 °C for 48 h. For the cytotoxicity test, the cells were challenged with MnFe_2_O_4_ nanoparticles (0, 20, 40, 80, 160 and 320 µg/mL) and incubated for 24, 48 and 72 h separately to study cell viability using 3-(4,5-dimethylthiazol-2-yl)-2,5-diphenyltetrazolium bromide (MTT) assay. About 10 mL of MTT solution (5 mg/mL) was added to each well and further kept for 4 h of incubation under the same conditions and using a multi-well ELISA plate reader, absorbance was recorded at 570 nm. The absorbance was converted to percentage of cell viability by using the following formula:

$$\%\;of\;cell\;viability\;=\frac{Value\;in\;the\;experimental\;sample}{Value\;of\;optical\;density\;in\;control\;sample}$$

### 3.4. Analysis of Apoptosis

#### 3.4.1. Acridine Orange/Propidium Iodide (AO/PI) Double Staining

Murine breast cancer cells (4T1) were plated at 1 × 10^6^ cells/well into 96-well plates and treated with various concentrations of MnFe_2_O_4_ nanoparticles (125, 250 and 500 µg/mL) for 24, 48 and 72 h independently. The cells were washed with PBS, fixed in methanol: acetic acid (3:1, *v*/*v*) for 10 min and stained with 10 μL of 10 mg/mL fluorescent dyes containing acridine orange (AO) and propidium iodide (PI) for 10 min. After washing the cells with phosphate buffer solution, stained cells were observed under FACS Calibur flow cytometry system (BD Biosciences, Franklin Lakes, NJ, USA).

#### 2.4.2. Annexin V-PI Staining

Apoptosis induction was studied in MnFe_2_O_4_ nanoparticle-challenged murine breast cancer cells (4T1) using annexin V-PI staining. Briefly, about 1 × 10^6^ cells/mL of murine breast cancer cells (4T1) were grown in 96-well plates and incubated for 24 h. Later, cells were challenged with different levels of MnFe_2_O_4_ nanoparticles (125, 250 and 500 µg/mL) and incubated for 24, 48 and 72 h. The cells were washed with phosphate buffer solution (1 mL) and centrifuged to get the cell pellet and later cells were added with binding buffer and stained with FITC-conjugated annexin V by following the manufacturer’s protocol (FITC Annexin V Apoptosis Detection Kit, BD Pharmingen, Franklin Lakes, NJ, USA). Using FACS Calibur flow cytometry system (BD Biosciences), the treated cells were visualized to record the data.

## 4. Conclusions

The sol-gel self-combustion method has been used for the successful preparation of manganese ferrite nanoparticles with an average particle size of about 30 nm. Face centered cubic MnFe_2_O_4_ nanoparticles were confirmed from the XRD result and was exactly matched with standard JCPDS data. The magnetic hysteresis properties such as saturation magnetization and coercivity at room temperatures were found to be 85 Am^2^/kg and 148 Gauss, respectively. MnFe_2_O_4_ nanoparticles exhibited dose dependent cytotoxic effects against 4T1 cells. Both apoptotic and necrotic activity were observed at all concentrations of MnFe_2_O_4_ nanoparticles tested. However, higher concentrations showed lesser cell survivability with increased necrosis. Our results suggest the possible exploration of MnFe_2_O_4_ nanoparticles as drug carrier agents in targeted cancer therapy.
